# Cerebral Microvascular and Systemic Effects Following Intravenous Administration of the Perfluorocarbon Emulsion Perftoran

**DOI:** 10.3390/jfb7040029

**Published:** 2016-11-18

**Authors:** Rania Abutarboush, Biswajit K. Saha, Saad H. Mullah, Francoise G. Arnaud, Ashraful Haque, Chioma Aligbe, Georgina Pappas, Charles R. Auker, Richard M. McCarron, Paula F. Moon-Massat, Anke H. Scultetus

**Affiliations:** 1 NeuroTrauma Department, Naval Medical Research Center (NMRC), Silver Spring, MD 20910, USA; biswajit.k.saha.ctr@mail.mil (B.K.S.); saad.h.mullah.ctr@mail.mil (S.H.M.); francoise.arnaud.ctr@mail.mil (F.G.A.); ashraful.haque.ctr@mail.mil (A.H.); chioma.aligbe@med.navy.mil (C.A.); georgina.pappas@quinnipiac.edu (G.P.); charles.auker.ctr@mail.mil (C.R.A.); richard.m.mccarron.civ@mail.mil (R.M.M.); pfmoonmassat@outlook.com (P.F.M.-M.); anke.h.scultetus2.ctr@mail.mil (A.H.S.); 2 Department of Surgery, Uniformed Services University of the Health Sciences, Bethesda, MD 20895, USA

**Keywords:** cerebral microcirculation, perfluorocarbon (PFC), oxygen therapeutic, vasoconstriction, intravital microscopy

## Abstract

Oxygen-carrying perfluorocarbon (PFC) fluids have the potential to increase tissue oxygenation during hypoxic states and to reduce ischemic cell death. Regulatory approval of oxygen therapeutics was halted due to concerns over vasoconstrictive side effects. The goal of this study was to assess the potential vasoactive properties of Perftoran by measuring brain pial arteriolar diameters in a healthy rat model. Perftoran, crystalloid (saline) or colloid (Hextend) solutions were administered as four sequential 30 min intravenous (IV) infusions, thus allowing an evaluation of cumulative dose-dependent effects. There were no overall changes in diameters of small-sized (<50 μm) pial arterioles within the Perftoran group, while both saline and Hextend groups exhibited vasoconstriction. Medium-sized arterioles (50–100 μm) showed minor (~8–9%) vasoconstriction within saline and Hextend groups and only ~5% vasoconstriction within the Perftoran group. For small- and medium-sized pial arterioles, the mean percent change in vessel diameters was not different among the groups. Although there was a tendency for arterial blood pressures to increase with Perftoran, pressures were not different from the other two groups. These data show that Perftoran, when administered to healthy anesthetized rats, does not cause additional vasoconstriction in cerebral pial arterioles or increase systemic blood pressure compared with saline or Hextend.

## 1. Introduction

Restoring tissue oxygenation following a hypoxic or ischemic event, such as hemorrhage, traumatic brain injury (TBI), or stroke, is critical for preventing hypoxia-induced pathophysiology and cell death. Over the past three decades, research focused on developing two classes of products as oxygen-carrying therapeutic fluids that augment circulating and tissue oxygenation: perfluorocarbon (PFC) emulsions and hemoglobin-based oxygen carriers (HBOC). Clinical side effects that include increased risk of myocardial infarction purportedly due to vasoconstriction and elevated mean and pulmonary blood pressures stalled clinical development of first- and second-generation HBOCs [1]. Small particle size and the interaction of cell-free hemoglobin in HBOCs with vascular endothelial cells leading to nitric oxide scavenging were proposed as mechanisms of vasoconstriction [2]. Chemical modifications led to a newer generation of oxygen therapeutics, including PFCs, which may offer the benefits of oxygen delivery to tissues and a lower incidence of vasoconstrictive side effects.

PFCs are synthetic and consist of a hydrophobic oxygen perfluorocarbon that is emulsified with surfactants and salts to make the product miscible with water and suitable for intravenous (IV) administration [3]. In the United States, the first-generation PFC Fluosol-DA^®^ (Green Cross Corp., Osaka, Japan) was approved by the Food and Drug Administration (FDA) for treatment of ischemia during balloon angioplasty in 1989. However, it was withdrawn from the market after five years due to its cumbersome preparation and pulmonary hypertensive effects [4]. Oxygent (Alliance Pharmaceutical Corp., San Diego, CA, USA) was a ‘second-generation’ commercial PFC that reached phase III clinical trials but failed to receive FDA approval for human use due to an increased incidence of strokes [5,6]. Currently, there are no PFCs approved for use as oxygen therapeutics in the United States. Perftoran was developed jointly by the Russian Academy of Sciences and the Perftoran Company (Pushchino, Russia) between 1980 and 1996 and is currently approved for clinical use in Russia and Mexico. The product is commercially registered as ‘Perftec’ in Mexico where it is manufactured by KEM Laboratory [7]. Perftoran is approved for human use for several indications, mainly as a substitute for blood during surgery, trauma and hemorrhagic shock; for treatment of various ischemic disorders including cerebral ischemia; and for organ preservation [8].

Perftoran consists of a 20% weight/volume (wt/vol) PFC emulsion of perfluorodecalin (PFD) and perfluoro-methylcyclohexylpiperidine (PFMCP) in a 2:1 ratio emulsified in Proxanol-268 [9]. The size of the PFC particles is 0.07 µm, approximately 1/100th of the size of erythrocytes [8]. The small particle size may, theoretically, allow Perftoran to perfuse narrowed vessels in the microcirculation better than erythrocytes and correct oxygen deficiencies after stroke, traumatic brain injury (TBI), or other microcirculatory disorders. Oxygen solubility in the PFC particles is 20 times higher than that in water or plasma. In vitro studies showed that treatment with Perftoran increased protein synthesis in cultured hepatic cells, presumably due to increased oxygenation [10]. In addition, Perftoran was clinically shown to decrease the need for blood transfusion and increase PaO_2_ without post-operative side effects in patients undergoing elective cardiac surgery [7]. The half-life of Perftoran in blood circulation is 24 h and the PFC components are mainly eliminated via the lungs, while Proxanol-268 is excreted by the kidneys within 24 h [11]. The LD_50_ in mice is 140 mL/kg and the permissible dose for humans is 20 mL/kg, as clinical observations at this dose have shown no hemolytic effects, no pyrogenic effects and no inhibition of hematopoiesis [11].

An ongoing effort of our laboratory is to identify and study potential treatments for TBI, specifically post-TBI decreased tissue oxygenation. In particular, it is of interest to assess this product for use as a treatment for TBI where the patient may or may not be normovolemic and, thus, clinically, the product could be given to a normovolemic patient as a “top-load” fluid, not as a blood substitute, but as an oxygen carrier. Since regulatory approval of previously developed oxygen therapeutics was hindered by concerns over their vasoconstrictive properties, the aim of this study was to assess the acute (150 min) vasoactive effects of Perftoran on cerebral pial microcirculation and its effect on systemic blood pressure in healthy rats. These effects were compared to two other commonly used but distinctly different classes of resuscitative fluids, crystalloids (i.e., normal saline) and colloids (i.e., Hextend) because Perftoran might have clinical application in the pre-hospital, far-forward military setting where these two alternative fluids are used. Hextend, in particular, has been used as a pre-hospital resuscitation fluid in the US military [12]. Since the half-life of Perftoran in circulation is 24 h, we used an escalation dose experimental design with doses that ranged from 5–20 mL/kg.

## 2. Results

### 2.1. Observations

All animals in the Perftoran (392 ± 34 g bodyweight (bw)), 0.9% NaCl (404 ± 52 g bw), and Hextend groups (404 ± 44 g bw) survived the entire study period (150 min) without clinical adverse events. Body temperatures did not change within or among the groups over the duration of the study. Mean body temperatures were 37.0 ± 0.3, 36.6 ± 0.5, and 36.9 ± 0.5 for Perftoran, 0.9% NaCl, and Hextend rats, respectively.

### 2.2. Pial Arteriolar Diameters

**Figure 1:**
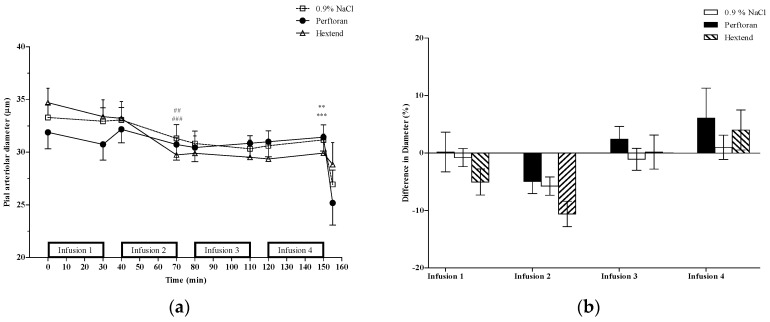
Small-sized pial arterioles (<50 µm). (**a**) Pial arteriolar diameters (mean ± SE) from rats treated with Perftoran (N = 51 arterioles from 13 rats), 0.9% NaCl (N = 71 arterioles from 13 rats), or Hextend (N = 46 arterioles from 12 rats). Test solutions were administered as four sequential 30 min IV infusions. The final data point for each treatment is vessel size after topical application of the vasoconstrictor, BaCl_2_ [13]. Statistically significant (*p* ≤ 0.05) change in vessel diameters comparing pre- vs. post-infusion values in the 0.9% NaCl (##), and Hextend (###) groups was observed at the end of the second infusion (T70). There was a significant cumulative change (T0 vs. T150) in vessel diameter within the 0.9% NaCl (**) and Hextend (***) groups; (**b**) Percent change of vessel diameters (mean ± SE) was determined using pre-infusion and post-infusion for each infusion separately. There were no differences in percent change among the three treatment groups for any of the infusions.

For small-sized pial arterioles (Figure 1), there was overall (comparing T0 to T150) cumulative vasoconstriction in both the 0.9% NaCl and Hextend groups as well as a significant (*p* < 0.05) vasoconstriction after the second infusion (T80 vs. T110) in both groups. There was no change in the Perftoran-treated group, either cumulatively or after any of the individual infusions (Figure 1).

**Figure 2:**
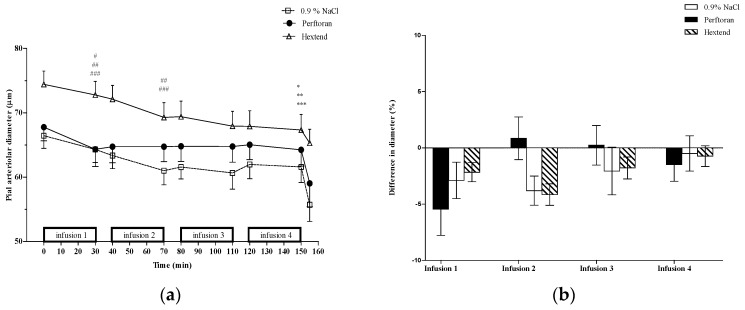
Medium-sized pial arterioles (50–100 µm). (**a**) Pial arteriolar diameters (mean ± SE) from rats treated with Perftoran (N = 34 arterioles from 13 rats), 0.9% NaCl (N = 56 arterioles from 13 rats), or Hextend (N = 51 arterioles from 12 rats). Test solutions were administered as four sequential 30 min IV infusions. The final data point for each treatment is vessel size after topical application of the vasoconstrictor BaCl_2_. Statistically significant (*p* ≤ 0.05) change in vessel diameters comparing pre- vs. post-infusion values in the Perftoran (#) group was observed at the end of the first (T30) infusion and at the end of the first (T30) and second (T70) infusions for 0.9% NaCl (##) and Hextend (###) groups. There was a significant cumulative change (T0 vs. T150) in vessel diameter within the Perftoran (*), 0.9% NaCl (**), and Hextend (***) groups; (**b**) Percent change of vessel diameters (mean ± SE) was determined using pre-infusion and post-infusion for each infusion separately. There were no differences in percent change among the three treatment groups for any of the infusions.

Over the course of the study (T0 vs. T150), there was vasoconstriction of medium-sized pial arterioles (Figure 2) in the Perftoran (5.3% ± 13.4%), 0.9% NaCl (9.3% ± 12.3%), and Hextend (8.1% ± 11.3%) groups, with Perftoran animals exhibiting smaller constriction than either of the other two treatment groups. Medium-sized vessels constricted equally in all of the groups after the first infusion (Figure 2). The second infusion of Perftoran caused no change in these vessels, while both 0.9% NaCl and Hextend infusions resulted in further vasoconstriction. There were no changes after the third and fourth infusions in these medium-sized arterioles in any of the treatment groups. However, for both small- and medium-sized vessels, there were no statistically significant differences in the percent change in the vessel diameter among the groups.

### 2.3. Heart Rate (HR) and Blood Pressures

HR was significantly different among the groups at baseline (T0) but was still within the normal range for rats under the anesthesia regimen used in this study [13,14,15,16,17] and did not exhibit any cumulative change (comparing T0 to T150) over time within any of the groups (Table 1). The only significant change in HR after an individual infusion was a slight transient increase in the 0.9% NaCl group after the third infusion. Among the groups, HR was significantly lower in the 0.9% NaCl group than the Perftoran group after each of the four infusions (i.e., T30, T70, T110, and T150) and was lower than the Hextend group after the first (T30) and third (T110) infusions. Heart rates in the Perftoran and Hextend groups were not different from each other at any point during the experiment.

**Figure 3:**
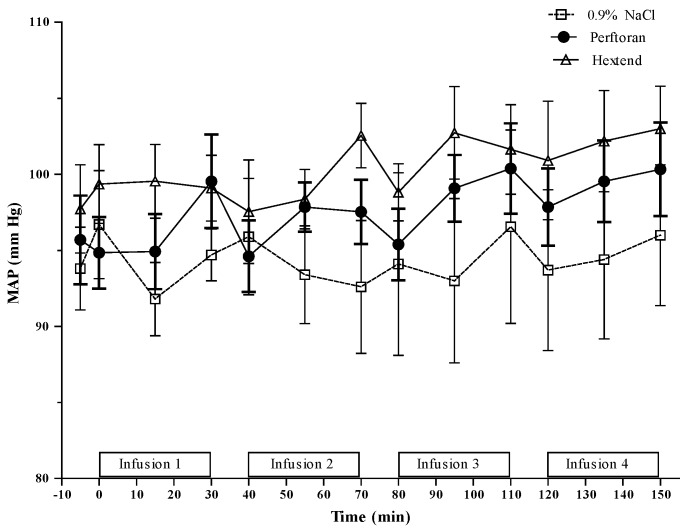
Mean arterial pressure (MAP). See Table 1 for statistical results of MAP.

Systolic (SBP), diastolic (DBP) and mean (MAP) arterial pressures for Perftoran, 0.9% NaCl and Hextend are presented in Table 1 and MAP data are also presented in Figure 3. There was an overall (T0 vs. T150) increase in arterial blood pressures in the Perftoran group, but not in the Hextend or 0.9% NaCl groups. This increase in arterial pressures, however, did not result in statistically significant differences between the Perftoran-treated rats and either of the treatment groups. In fact, among the groups, the only significant differences in MAP, SBP and DBP were between the Hextend and 0.9% NaCl groups at the end of the second infusion (T70), where arterial blood pressures were, as expected, significantly higher in the Hextend group.

Within the groups, MAP increased after the third and fourth infusions in the Perftoran-treated rats, resulting in a 7 mm Hg overall increase. SBP values in the Perftoran group were significantly higher than the pre-infusion values at the end of the first (T30) and third (T110) infusions. These changes resulted in a 10 mm Hg cumulative increase in SBP. At T150, DBP values had increased by 6 mm Hg compared to T0 baseline values.

### 2.4. Arterial Blood Gas Parameters

In spite of occasional statistically significant changes in blood parameters, overall measurements were within the normal ranges for rats (Table 2). There was an overall decrease in pH in Perftoran-treated rats with lower pH values at the end of each infusion compared to the T0 value; however, pH values in Perftoran rats were not statistically different from the two other treatment groups. The decrease in pH is probably due to metabolic changes or the mild increase in PaCO_2_ within the Perftoran group. HCO_3_^−^ decreased over time while lactate values remained constant in the Perftoran rats, ruling out lactate buildup as a cause of the observed decrease in pH. Total Hb values were expectedly lower in both the Perftoran and Hextend rats than the rats that received 0.9% NaCl due to the larger infusion volume administered to those rats. There were statistically significant differences among the groups in HCO_3_^−^ due to higher bicarbonate in the Hextend group and, therefore, these differences were not relevant to Perftoran treatment effects.

## 3. Discussion

This study demonstrated that the PFC emulsion Perftoran did not cause acute vasoconstriction in cerebral pial arterioles compared to 0.9% NaCl (normal saline)- and Hextend-treated animals in a healthy anesthetized rat cranial window model. It is important to note that this similarity in the three fluids was observed despite the large differences in their fluid characteristics and the unequal rates of administration. The infusion rates of the comparator fluids were selected to “bracket” possible effects on hemodynamic parameters and pial vessel diameters. The intravascular retentions of crystalloids (0.9% NaCl) and colloids (Hextend) are different, with 0.9% NaCl translocating into the extravascular space rapidly and Hextend being retained in the circulation for a longer duration. The maintenance infusion of 0.9% NaCl (4 mL/kg/h to replace insensible fluid losses) was expected to have the least effect upon the animals, while a higher rate of infusion of the colloid Hextend (8 mL/kg/h) was expected to have the largest effect. Hextend is a powerful plasma expander with a colloid osmotic pressure (COP) of ~36 mm Hg and is used in the US military at a dose similar to that used in this study in pre-hospital and far-forward battle environments as a resuscitation fluid. Since Perftoran lacks an oncotic agent and, thus, has negligible COP similar to 0.9% NaCl [9], it is presumed to behave more like a crystalloid fluid than a colloid. Perftoran was administered at the manufacturer’s recommended rate of 10 mL/kg/h. Thus, at the administered doses, the effect on volume expansion was expected to be highest for Hextend and lowest for saline, with Perftoran somewhere in between. Compared to both of these fluids, Perftoran showed no additional vasoconstriction of pial vessels, a critical safety concern for this product if used for TBI.

In medium-sized pial microvessels, infusion of 0.9% NaCl, Hextend, and Perftoran caused minor vasoconstriction within each of their respective groups over time but there were no differences among the groups. In contrast, small-sized vessels did not exhibit vasoconstriction following Perftoran infusion, but did show overall constriction with 0.9% NaCl and Hextend infusions. This trend following saline infusion was previously reported by our laboratory [13] and it is most likely associated with the cranial window model and may be a result of the anesthesia, surgical instrumentation (craniotomy and cutting the dura), localized changes in the surface brain temperature, or intracranial pressure following removal of the dura. Since none of the treatment solutions were heated before being infused, it is also possible that these small changes in vessel diameter may be due, in part, to small changes in blood temperature, and while this is unlikely since the solutions were administered slowly and in small volumes and were therefore thoroughly mixed with warm blood before reaching the brain, it cannot be ruled out.

The effects of Perftoran infusion on the diameters of cerebral pial arterioles reported here are similar to those reported by Eckmann and Lomivorotov [18] using the rat cremaster muscle microcirculation to evaluate the use of Perftoran for the clearance of air embolism. In their study, the administration of Perftoran as a bolus over 5 min did not cause any changes in microvessel diameter or tone, defined as the ability to constrict or dilate when phenylephrine or acetycholine, respectively, were topically applied to the muscle. In another intravital microscopy study that examined the effects of PFC on intestinal ischemia/reperfusion, treatment with Perftoran reduced the observed constriction in rat mesenteric arteries during the reperfusion period by ~20% [19]. To our knowledge, the present study is the first to assess the vasoactive effects of Perftoran on cerebral vasculature. Collectively, these three intravital microscopic studies (including this report) show that Perftoran lacks vasoconstrictive properties in skeletal muscle, smooth muscle and the pial microcirculation.

In the present study, there were no differences in systemic blood pressures or HR between Perftoran and the 0.9% NaCl or Hextend groups, in spite of a modest (~6–10 mm Hg) increase in arterial blood pressures within the Perftoran group. This would indirectly suggest that the product does not cause systemic vasoconstriction; however, more invasive monitoring (e.g., cardiac output and pulmonary pressures, among other parameters) is necessary to better characterize the effects of Perftoran on hemodynamics. Similar to our findings, Eckmann and Lomivorotov [18] reported no changes in arterial blood pressures or heart rate compared to their control animals with the administration of 6.4 mL/kg of Perftoran as a bolus over 5 min to normovolemic rats. In addition, when patients in a clinical trial received two 2.5 mL/kg infusions of Perftoran, resulting in a total cumulative dose of 5 mL/kg (which is equivalent to the first dose of our study) during an acute normovolemic hemodilution protocol prior to cardiac surgery, there were no differences in arterial blood pressures, HR, respiratory rate, or central venous blood pressure in the Perftoran group compared to the control (standard care) group that received crystalloid infusions [7]. Even though the cumulative dose of Perftoran used in our study (20 mL/kg) was four times the dose used in the previously mentioned clinical trial, there were no significant differences in blood pressure among the Perftoran and the colloid and crystalloid groups, suggesting hemodynamic stability even with higher doses. The observed statistically significant increase in arterial blood pressures within the Perftoran group may not be physiologically significant for two reasons: (1) the values were within normal range and (2) none of the values were statistically higher than those observed in the saline or colloid animals and, in fact, the Perftoran SBP values at the end of each infusion were always lower than those of the colloid (Hextend)-treated animals. The higher HR values in the Perftoran group compared to the 0.9% NaCl group were a result of the statistically higher HR values in the Perftoran group at baseline.

The arterial blood gas and hemoglobin data of animals in this study were within expected values. Although the observed decrease in pH within the Perftoran group was not statistically different from the 0.9% NaCl or Hextend animals, it is still inconsistent with clinical findings that report diminishment of acidosis in patients treated with Perftoran [8]. The reasons behind this inconsistency include species and “application” differences. Specifically, the cited clinical studies were in patients that experienced blood loss and Perftoran was used for volume replacement, maintenance of arterial PaO_2_, and prevention of hypoxia and ischemia, while in our animal model the product was administered to normovolemic animals that did not experience any blood loss.

Several studies indicate that Perftoran reduces the effects of ischemia and hypoxia, presumably due to its oxygen-carrying properties. In a study of 117 kidney grafts (58 initially perfused with Perftoran vs. 59 controls), Reznik et al. [20] showed improved tissue preservation and decreased ischemia/reperfusion injury of the kidney graft using Perftoran, as measured by time to transplant function (twice as fast in the Perftoran patients). Also, enhanced oxygenation with Perftoran of a bio-artificial liver (developed to assist patients with acute liver failure) resulted in a significant increase in protein synthesis by human hepatic cells in vitro [10]. Treatment with Perftoran decreased the need of allogeneic blood transfusions in patients undergoing cardiopulmonary bypass [7]. In a review of the Russian scientific literature, Maevsky and coauthors [8] concluded that increased tissue oxygenation with Perftoran treatment was not only useful in blood loss cases, but also in various vessel occlusive diseases (i.e., cardiac infarct), burns, and TBI. The Perftoran doses used in these studies ranged from 4–30 mL/kg with lower doses (4–6 mL/kg) suggested to be most beneficial to TBI patients [8]. Both our cumulative dose (20 mL/kg) and our first infusion dose (5 mL/kg) fit within these two dose ranges.

Due to the paucity of data on the specific effects of Perftoran treatment on TBI, further studies are needed to show whether Perftoran is capable of increasing brain tissue oxygenation after TBI and whether early treatment with the PFC could improve neurocognitive and histological outcomes post-TBI. Our study showed that Perftoran lacks cerebral vasoconstrictive effects as measured by direct evaluation of pial arteriolar diameters before and after administration of a cumulative dose of 20 mL/kg of the PFC to healthy, uninjured, normovolemic, and hemodynamically stable animals. Further studies to determine therapeutic efficacy in a TBI model are indicated.

## 4. Materials and Methods

The study protocol was reviewed and approved by the Walter Reed Army Institute of Research/Naval Medical Research Center Institutional Animal Care and Use Committee in compliance with all applicable Federal regulations governing the protection of animals in research.

*Test solutions.* Perftoran was obtained from Perftoran Research and Production Company (Moscow, Russia). Before use, the emulsion was mixed thoroughly by inverting and righting the tube 10–15 times until the mixture appeared homogenous and no product separation or precipitate were visible. Perftoran was administered as four 30 min infusions separated by 10 min at a rate of 10 mL/kg/h per infusion for a cumulative volume of 20 mL/kg, the highest manufacturer’s estimated recommended dose for human use.

The two most commonly used asanguineous clinical fluid types, a colloid and a crystalloid, served as comparators for the Perftoran data. The crystalloid group received normal saline (0.9% NaCl; Abbott Laboratories, Chicago, IL, USA) at a maintenance rate of 4 mL/kg/h, given to replace insensible fluid loses and counteract some depressant effects of anesthesia. It was postulated that Perftoran would behave like 0.9% NaCl, a crystalloid fluid, as it lacks an oncotic agent and thus has negligible colloid osmotic pressure (COP) [9]. However, because Perftoran is not a water-soluble mixture and the COP of Perftoran is unknown, we included the colloid group. The colloid group received 6% hetastarch in lactated Ringer’s solution (Hextend; Hospira, Lake Forest, IL, USA) at 8 mL/kg/h. We administered this high dose of colloid to allow a “bracketing” of Perftoran with these two treatment groups. In addition, Hextend was the colloid of choice because it has been used as pre-hospital resuscitation fluid when blood is not available in the US military at a similar dose [12].

*Animal preparation.* Male Sprague-Dawley rats weighing 300–450 g were anesthetized with ketamine/acepromazine (72/4 mg/kg, respectively, intraperitoneal). A femoral vein was cannulated with PE-50 catheter for infusion of the test solutions. A femoral artery was cannulated (PE-50) for blood sampling and arterial blood pressure and heart rate monitoring (Datascope Corporation, Montvale, NJ, USA). All animals were mechanically ventilated (small animal ventilator, Kent Scientific, Lichfield, CT, USA) using 40% oxygen and 60% nitrogen at settings to maintain normoxia (PaO_2_ > 100 mmHg) and normocapnia (35–45 mm Hg). 

A cranial window was prepared as previously described [13,14,17]. Briefly, pial microvasculature was accessed through a rectangular craniotomy prepared in the right parietal bone. The dura was cut and reflected to the side to allow clear visualization of pial surface microvessels. The surface of the brain was superfused with artificial cerebrospinal fluid (aCSF; 150 mM Na, 3.0 mM K, 1.4 mM Ca, 0.8 mM Mg, 1.0 mM P, 155 mM Cl; Harvard Apparatus, Holliston, MA, USA) to maintain electrolyte balance. The pO_2_ and pCO_2_ were not measured. The aCSF was warmed up to 37 °C at the beginning of the experiment. A stereomicroscope (SZ16, Olympus, Tokyo, Japan) equipped with a lens with a numerical aperture (NA) of 0.15 and a DP-73 digital camera was used for direct visualization and imaging of pial microvessels at a total magnification of 80× and 0.8063 pixel/micrometer for both X and Y dimensions. 

*Experimental protocol.* After surgical preparation, a 15 min stabilization period was allowed and baseline (T0) data were collected. Rats were then randomized to the experimental (Perftoran, N = 13), crystalloid (0.9% NaCl, N = 13) or colloid (Hextend, N = 12) groups. Each animal received four sequential IV infusions based on their treatment group, each infusion lasting 30 min, with 10 min between infusions. Designing the protocol with these four measurements times during the 150 min study allowed an evaluation of four dose-dependent effects (5, 10, 15 and 20 mL/kg Perftoran) over the full experiment.

Pial arteriolar vessel diameters, heart rate (HR), body temperature, and hemodynamic parameters (systolic, diastolic and mean arterial pressures) were recorded at the beginning and end of each infusion. Arterial blood samples, obtained at baseline and at the end of each infusion, were analyzed on an automated blood gas system (ABL 700, Radiometer, Copenhagen, Denmark) for hemoglobin (Hb), pH, partial pressures of oxygen (PaO_2_) and carbon dioxide (PaCO_2_), lactate, and bicarbonate (HCO_3_^−^).

In each animal, pial arteriolar microvessel diameters were measured at the same locus using the program CellSens (Olympus, Tokyo, Japan, 2010). At the end of the observation period, a few drops of 5% aqueous barium chloride (BaCl_2_) were topically applied to pial arterioles to confirm vessel responsiveness as BaCl_2_ is a strong vasoconstrictor in cerebral blood vessels [21].

*Statistical Analyses.* Mixed general linear models were used for analyzing heart rate, blood pressure, vessel diameter, temperature, blood gases, Hb, and lactate data, while rat body weights were analyzed using a one-way analysis of variance (ANOVA). The amount of smooth muscle increases with vessel size which could lead to differences in degree of vessel contraction [21], therefore and as previously reported in other cerebrovascular studies [13,22,23], pial arterioles were divided into three categories based on vessel diameter at baseline measurement: (1) small (<50 µm); (2) medium vessels (50–100 µm); and (3) large (>100 µm). For the mixed model, the fixed effects in the ANOVA were group (PFC, Hextend, and 0.9% NaCl) and observation period (i.e., T0, T30, T70, T80, T110, T120, and T150). This test was used to determine whether the differences among the groups varied as a function of treatment and observation period. Following this analysis, a one-way repeated measures ANOVA was performed to determine whether the measurement changed over time within each treatment group. For vessel diameters, mean percent change from baseline was compared between pairs of treatment groups. Percent change was determined using pre- and post-infusion data for each infusion separately. Least square differences were used for post hoc *t*-tests. A *p*-value ≤ 0.05 was considered statistically significant for all parameters. IBM SPSS Statistics 21.0 (IBM Corporation, Armonk, NY, USA, 2012) software was used for data analyses.

## 5. Conclusions

This study demonstrated that the oxygen-carrying perfluorocarbon emulsion Perftoran lacks cerebral vasoactive effects and has an acceptable hemodynamic profile as compared to saline- and colloid-treated animals in a healthy normovolemic rat model. Perftoran should be evaluated further to determine if there is clinical efficacy and if it is otherwise safe in a relevant, large animal TBI model.

## Figures and Tables

**Table 1:** Blood pressure and heart rate parameters for Perftoran, Hextend, and 0.9% NaCl groups. All values are means ± SE. *: Post-infusion value (T30, T70, T110 or T150) is significantly different (*p* ≤ 0.05) from its corresponding within-group value at T0; values at pre-infusion times T40, T80, and T120 were not compared to T0. #: Post-infusion value (T30, T70, T110 or T150) was significantly different from its corresponding within-group value at the preceding pre-infusion time point (T0, T40, T80, or T120). Statistically significant differences among the groups at each time point are marked with similar superscripted letters (a or b).

Time	Treatment	HR (Beats/min)	SBP (mm Hg)	MAP (mm Hg)	DBP (mm Hg)
T0 (baseline)	Perftoran	415 ± 20 ^a^	122 ± 4	95 ± 2	79 ± 2
	Hextend	392 ± 25 ^b^	129 ± 3	99 ± 3	82 ± 3
	0.9% NaCl	305 ± 17 ^a,b^	119 ± 4	94 ± 4	79 ± 3
T30 (after infusion 1)	Perftoran	406 ± 18 ^a^	129 ± 4 ^#,^*	100 ± 3	82 ± 3
	Hextend	367 ± 24 ^b^	130 ± 3	99 ± 2	81 ± 3
	0.9% NaCl	308 ± 12 ^a,b^	121 ±2	93 ± 2	77 ± 2
T40 (before Infusion 2)	Perftoran	406 ± 25	124 ± 4	95 ± 2	78 ± 1
	Hextend	350 ± 30	126 ± 4	98 ± 3	80 ± 4
	0.9% NaCl	338 ± 10	122 ± 4	95 ± 4	79 ± 3
T70 (after Infusion 2)	Perftoran	388 ± 20 ^a^	129 ± 4 *	98 ± 2	81 ± 2
	Hextend	364 ± 24	133 ± 4 ^a^	103 ± 2 ^a^	85 ± 1 ^a^
	0.9% NaCl	318 ± 13 ^a^	121 ± 4 ^a^	92 ± 4 ^a^	77 ± 4 ^a^
T80 (before Infusion 3)	Perftoran	409 ± 17	125 ± 4	95 ± 2	79 ± 2
	Hextend	378 ± 23	128 ± 3	99 ± 2	81 ± 2
	0.9% NaCl	336 ± 20	119 ± 5	93 ± 5	77 ± 5
T110 (after Infusion 3)	Perftoran	390 ± 17 ^a^	132 ± 5 ^#,^*	100 ± 3 *	84 ± 3
	Hextend	388 ± 26 ^b^	132 ± 4	102 ± 3	84 ± 2
	0.9% NaCl	331 ± 14 *^,a,b^	124 ± 5	94 ± 5	79 ± 5
T120 (before Infusion 4)	Perftoran	389 ± 23	129 ± 4	98 ± 3	82 ± 2
	Hextend	371 ± 23	132 ± 5	101 ± 4	83 ± 3
	0.9% NaCl	351 ± 14	118 ± 5	92 ± 3	75 ± 3
T150 (after Infusion 4)	Perftoran	386 ± 21 ^a^	132 ± 5 *	100 ± 3 *	84 ± 3 *
	Hextend	364 ± 21	135 ± 5	103 ± 3	85 ± 2
	0.9% NaCl	325 ± 17 ^a^	125 ± 4	94 ± 4	77 ± 4

**Table 2:** Arterial blood parameter analysis (mean ± SE). *: Indicates that this measurement (T30, T70, T110 or T150) was significantly (*p* ≤ 0.05) different from T0 within a treatment group. Treatment points with similar superscript letters (a or b) were statistically different from each other.

Time	Group	Total Hb (g/dL)	pH	PaCO_2_ (mm Hg)	PaO_2_ (mm Hg)	HCO_3_ (mmol/L)	Lactate (mmol/L)
T0 (baseline)	Perftoran	11.7 ± 0.3	7.377 ± 0.023	41.4 ± 3.1	128.4 ± 34.4	23.1 ± 0.5	0.6 ± 0.1
	Hextend	12.5 ± 0.3	7.355 ± 0.014	45.4 ± 2.1	131.4 ± 22.9	24.0 ± 0.6	0.5 ± 0.1
	0.9% NaCl	12.5 ± 0.3	7.349 ± 0.022	46.9 ± 3.2	109.6 ± 11.3	23.5 ± 0.3	0.6 ± 0.1
T30 (after Infusion 1)	Perftoran	11.5 ± 0.3 ^a^	7.355 ± 0.020 *	43.8 ± 3.2	139.9 ± 37.6	22.7 ± 0.3 ^a^	0.5 ± 0.1
	Hextend	11.5 ± 0.3	7.367 ± 0.019	43.8 ± 2.8	134.1 ± 24.1	23.7 ±0.3 ^a^	0.6 ± 0.1
	0.9% NaCl	12.4 ± 0.3 ^a^	7.352 ± 0.018	45.5 ± 2.7	115.7 ± 9.5	23.4 ± 0.3	0.7 ± 0.1
T70 (after Infusion 2)	Perftoran	11.3 ± 0.3 ^a^	7.356 ± 0.021 *	42.3 ± 3.1	135.0 ± 34.1	22.3 ± 0.4	0.6 ± 0.1
	Hextend	11.0 ± 0.3 ^b^	7.364 ± 0.015	42.5 ± 2.2	131.6 ± 21.9	23.1 ± 0.5	0.6 ± 0.1
	0.9% NaCl	12.3 ± 0.4 ^a,b^	7.354 ± 0.019	42.3 ± 2.6	117.5 ± 8.5	22.4 ± 0.4	0.7 ± 0.1
T110 (after Infusion 3)	Perftoran	11.3 ± 0.3 ^a^	7.348 ± 0.019 *	42.8 ± 3.0	135.4 ± 39.4	22.2 ±0.4 ^a^	0.5 ± 0.1
	Hextend	10.8 ± 0.3 ^b^	7.347 ± 0.010	45.3 ± 2.0	134.0 ± 24.0	23.3 ±0.4 ^a,b^	0.8 ± 0.1 *
	0.9% NaCl	12.1 ± 0.3 ^b^	7.340 ± 0.016	42.7 ± 2.6	110.6 ± 8.9	21.8 ± 0.4 ^b^	0.7 ± 0.1
T150 (after Infusion 4)	Perftoran	11.7 ± 0.3	7.338 ± 0.020 *	45.6 ± 3.5 *	147.4 ± 39.1	22.5 ± 0.4 ^a^	0.6 ± 0.1 ^a^
	Hextend	11.1 ± 0.2 *^,b^	7.336 ± 0.011	48.5 ± 2.4	125.1 ± 21.1	23.8 ±0.5 ^a,b^	1.0 ± 0.1 *^,a^
	0.9% NaCl	12.6 ± 0.3 ^a,b^	7.323 ± 0.014 *	46.6 ± 2.2	105.6 ± 8.4	22.2 ± 0.3 ^b^	0.7 ± 0.1

## References

[1] Natanson C., Kern S.J., Lurie P., Banks S.M., Wolfe S.M. (2008). Cell-free hemoglobin-based blood substitutes and risk of myocardial infarction and death—A meta-analysis. JAMA.

[2] Olson J.S., Foley E.W., Rogge C., Tsai A.L., Doyle M.P., Lemon D.D. (2004). No scavenging and the hypertensive effect of hemoglobin-based blood substitutes. Free Radic. Biol. Med..

[3] Riess J.G. (2005). Understanding the fundamentals of perfluorocarbons and perfluorocarbon emulsions relevant to in vivo oxygen delivery. Artif. Cells Blood Substit. Biotechnol..

[4] Vercellotti G., Hammerschmidt D., Craddock P., Jacob H. (1982). Activation of plasma complement by perfluorocarbon artificial blood: Prob-able mechanism of adverse pulmonary reactions in treated patients and rationale for corticosteroids prophylaxis. Blood.

[5] Spahn D.R., Waschke K.F., Standl T., Motsch J., Van Huynegem L., Welte M., Gombotz H., Coriat P., Verkh L., Faithfull S. (2002). Use of perflubron emulsion to decrease allogeneic blood transfusion in high-blood-loss non-cardiac surgery: Results of a european phase 3 study. Anesthesioloy.

[6] Palmer A.F., Intaglietta M. (2014). Blood substitutes. Annu. Rev. Biomed. Eng..

[7] Verdin-Vasquez R.C., Zepeda-Perez C., Ferra-Ferrer R., Chavez-Negrete A., Contreras F., Barroso-Aranda J. (2006). Use of perftoran emulsion to decrease allogeneic blood transfusion in cardiac surgery: Clinical trial. Artif. Cells Blood Substit. Biotechnol..

[8] Maevsky E., Ivanitsky G., Bogdanova L., Axenova O., Karmen N., Zhiburt E., Senina R., Pushkin S., Maslennikov I., Orlov A. (2005). Clinical results of perftoran application: Present and future. Artif. Cells Blood Substit. Biotechnol..

[9] Maevsky E.I., Ivanitsky H.R., Islamov B.I., Moroz V.V., Bogdanova L.A., Karmen N.B., Pushkin S.Y., Maslennikov I.A., Winslow R.M. (2006). Perftoran. Blood Substitutes.

[10] Kinasiewicz A., Smietanka A., Gajkowska B., Werynski A. (2008). Impact of oxygenation of bioartificial liver using perfluorocarbon emulsion perftoran on metabolism of human hepatoma C3A cells. Artif. Cells Blood Substit. Immobil. Biotechnol..

[11] KEM Laboratories (2012). Perftec: A Blood Substitute with Oxygen Transporting Capacity.

[12] Butler F.K., Schreiber M.A., Kotwal R.S., Jenkins D.A., Champion H.R., Bowling F., Cap A.P., Dubose J.J., Dorlac W.C., Dorlac G.R. (2014). Fluid resuscitation for hemorrhagic shock in tactical combat casualty care: Tccc guidelines change 14-01—2 June 2014. J. Spec. Oper. Med..

[13] Abutarboush R., Scultetus A., Arnaud F., Auker C., McCarron R., Moon-Massat P.F. (2013). Effects of *N*-acetyl-l-cysteine and hyaluronic acid on HBOC-201-induced systemic and cerebral vasoconstriction in the rat. Curr. Drug Discov. Technol..

[14] Abutarboush R., Aligbe C., Pappas G., Saha B., Arnaud F., Haque A., Auker C., McCarron R., Scultetus A., Moon-Massat P. (2014). Effects of the oxygen-carrying solution oxyvita C on the cerebral microcirculation and systemic blood pressures in healthy rats. J. Funct. Biomater..

[15] Mullah S.H., Abutarboush R., Moon-Massat P.F., Saha B.K., Haque A., Walker P.B., Auker C.R., Arnaud F.G., McCarron R.M., Scultetus A.H. (2016). Sanguinate’s effect on pial arterioles in healthy rats and cerebral oxygen tension after controlled cortical impact. Microvasc. Res..

[16] Mullah S.H., Saha B.K., Abutarboush R., Walker P.B., Haque A., Arnaud F.G., Hazzard B., Auker C.R., McCarron R.M., Scultetus A.H. (2016). Perfluorocarbon NVX-108 increased cerebral oxygen tension after traumatic brain injury in rats. Brain Res..

[17] Moon-Massat P., Mullah S.H., Abutarboush R., Saha B.K., Pappas G., Haque A., Auker C., McCarron R.M., Arnaud F., Scultetus A. (2015). Cerebral vasoactivity and oxygenation with oxygen carrier M101 in rats. J. Neurotrauma.

[18] Eckmann D.M., Lomivorotov V.N. (2003). Microvascular gas embolization clearance following perfluorocarbon administration. J. Appl. Physiol..

[19] Kozhura V.L., Basarab D.A., Timkina M.I., Golubev A.M., Reshetnyak V.I., Moroz V.V. (2005). Reperfusion injury after critical intestinal ischemia and its correction with perfluorochemical emulsion ”perftoran”. World J. Gastroenterol..

[20] Reznik O.N., Bagnenko S.F., Loginov I.V., Iljina V.A., Ananyev A.N., Moysyuk Y.G. (2008). The use of oxygenated perfluorocarbonic emulsion for initial in situ kidney perfusion. Transplant. Proc..

[21] Rosenblum W.I. (1976). Pial arteriolar responses in mouse-brain revisited. Stroke.

[22] Rebel A., Cao S., Kwansa H., Doré S., Bucci E., Koehler R.C. (2006). Dependence of acetylcholine and ADP dilation of pial arterioles on heme oxygenase after transfusion of cell-free polymeric hemoglobin. Am. J. Physiol. Heart Circ. Physiol..

[23] Rebel A., Ulatowski J.A., Kwansa H., Bucci E., Koehler R.C. (2003). Cerebrovascular response to decreased hematocrit: Effect of cell-free hemoglobin, plasma viscosity, and CO_2_. Am. J. Physiol. Heart Circ. Physiol..

